# Decisions to use surgical mesh in operations for pelvic organ prolapse: a question of geography?

**DOI:** 10.1007/s00192-018-3788-y

**Published:** 2018-10-20

**Authors:** Emil Karl Nüssler, Emil Nüssler, Jacob Kjær Eskildsen, Mats Löfgren

**Affiliations:** 1 Department of Clinical Science, Obstetrics and Gynecology, Umeå University, Umeå, Sweden; 2 Gynop-registret, Norrlands universitetssjukhus, 90185 Umeå, Sweden; 3 Department of Management, School of Business and Social Sciences, Aarhus University, Aarhus, Denmark

**Keywords:** Quality control, Surgical decision-making, Surgical mesh, Surgical learning

## Abstract

**Introduction and hypothesis:**

Surgical mesh can reinforce damaged biological structures in operations for genital organ prolapse. When a method is new, scientific information is often contradictory. Individual surgeons may accept different observations as useful, resulting in conflicting treatment strategies. Additional scientific information should lead to increasing convergence.

**Methods:**

Based on data from the Swedish National Quality Register of Gynecological Surgery, all patients who underwent their first recurrent anterior compartment prolapse operation between 2006 and 2017 were included (2758 patients). Surgical mesh was used in 56.5%. We analyzed inter-county disparities in and patterns of mesh use over 12 years. To minimize confounding, we selected a group of highly comparable patients where similar decision patterns could be expected.

**Results:**

The use of mesh differed between counties by a factor of 11 (8.6–95.3%). Counties with low use of mesh continued with low use and counties with high use continued with high use.

**Conclusions:**

Decisions regarding how to interpret existing scientific information about mesh implants in the early years of mesh use have led to “communities of practice” highly influenced by geographical factors. For 12 years, these groups have made disparate decisions and upheld them without measurable change toward consensus. The scientific learning process has stopped—despite the abundance of new publications and the steady supply of new types of mesh. Ongoing disparity in surgeons’ choices in comparable patients has an adverse effect on clinical care. For the patient, this represents 12 years of a geographical lottery concerning whether mesh is used or not.

## Introduction

Pelvic organ prolapse (POP) is common in women. Approximately 12% of women undergo an operation for prolapse [1–3], and high rates of recurrence in the range of 30–40% have been reported [4, 5]. To provide additional support for weakened or damaged biological tissue, special surgical meshes have been designed and have been in use since the US Food and Drug Administration (FDA) approved the first mesh products in 2002 [6]. In contrast to the stringent requirements and formalized approaches for development of pharmaceuticals, there has never been a systematic scientific premarketing evaluation of mesh products. Faced with this situation, every surgeon individually has to investigate and validate available scientific information and accept the information gleaned as "current best knowledge." Consequently, treatment of POP with mesh remains controversial, and the decision when to use mesh is an unresolved challenge for gynecological surgeons and patients [7]. In 2011, the FDA released an update regarding the use of transvaginal surgical mesh, including a warning because of “serious safety and effectiveness concerns” [6].

A recent article [8] investigated 684,250 POP procedures performed in 2012 in 15 Organization for Economic Co-operation and Development (OECD) countries including Sweden. The article shows an extraordinary lack of uniformity across these 15 countries, with the median rate of surgical mesh utilization in the anterior compartment differing by a factor of 7.9 (range 3.3–26%) and in the posterior compartment by a factor of 5.3 (range 3.3–17.0%).

Monitoring results in prolapse surgery, the Swedish National Quality Register of Gynecological Surgery (GynOp) has seen an even larger continuing disparity in mesh use in Sweden.

The optimal rate of mesh use cannot be both low and high at the same time. A large and persisting geographical disparity in mesh use in comparable patient groups will affect clinical care negatively.

The aim of this article is: (1) to describe the geographical disparity over 12 years in the use of surgical mesh in operations for POP in Sweden; (2) to chart changes in patterns of Swedish gynecological surgeons’ mesh use over time.

## Methods

### The Swedish National Quality Register of Gynecological Surgery

The GynOp register includes all major gynecological operations performed in Sweden. Since 2006, GynOp has registered prolapse operations in detail on a national scale, including a 1-year follow-up of patients. Today, the register contains complete information on more than 56,000 prolapse procedures [9]. All patients are included in the register when a urogynecological procedure is decided. Yearly comparisons with the Swedish National Patient Register (where all Swedish surgical procedures are registered by law) shows that the GynOp coverage of Swedish prolapse operations from 2006 to 2008 has been around 75% and since 2009 it has continuously been > 95%. The data collection process includes both surgeon and patient-derived data up to 1-year post-operation [10–12]. Data completeness regarding the use of mesh reported by the surgeons has been 100%. The GynOp registry provides all Swedish gynecological surgeons with yearly reports that include detailed information about the use of mesh in all types of prolapse surgery.

### The Swedish hospital system

The Swedish hospital system is primarily organized at a county level. Swedish counties are fairly independent political units responsible for all health services within their boundaries [13]. Public hospitals are owned by the counties and financed by county taxes. There are a few private clinics that specialize primarily in elective surgery. These clinics are contracted to county councils and reimbursed by them for operations they perform on Swedish patients as all Swedes are covered under the national health system.

In an effort to make the hospital system more efficient, some counties have supported specialization in some hospitals, so differences in use of mesh at a hospital level may in some cases have organizational rather than medical reasons.

The GynOp register shows that practically all (98.2%) patients who undergo a prolapse operation are operated on in the county they live in. This makes county results useful and robust as parameters for analysis of changes in mesh use. Based on this fact, we hypothesized that the proportion of POP operations with mesh county-wise, stratified by years, expresses the particular mesh policy for a particular year.

### Data

The basic data used in this study include all POP operations registered prospectively and consecutively in GynOp from 1 January 2006 to 29 August 2017—in all, 56,120 operations. Patients with simultaneous operations for incontinence were not included. To minimize confounding, we selected a cohort in which the use of mesh was an accepted alternative and whose patients were so comparable that similar mesh decisions could be expected for them.

We included only (1) patients who underwent only operations in the anterior compartment (anterior colporrhaphy). This is the most common operation in prolapse surgery and has a moderate level of difficulty. Patients with concomitant POP or non-POP operations were excluded. Additionally, (2) only healthy patients were included in the study (American Society of Anesthesiologists’ classification system for patients’ preoperative physical status, group one or two) [14]. Moreover, (3) all selected patients had a normal non-descended uterus. Since it can be argued that primary and recurrent POP operations represent different non-comparable patient groups, we (4) analyzed only patients undergoing their first recurrent operation, where the use of mesh is a generally accepted option; 96.7% of these patients had previously undergone “native tissue repair” operations and 3.3% had received a mesh.

We excluded four small counties and/or counties with low activity regarding POP surgery that reported fewer than 50 recurrent operations each (in total, 103 patients). This rigorous selection process resulted in a study group of 2758 eligible patients with recurrent POP surgery in the anterior compartment, operated on by 467 Swedish gynecological surgeons in 52 gynecological departments in 17 counties over 12 years where surgical mesh was used in 1559 patients (56.5%). All statistical analyses were performed using SPSS version 23 (IBM, Armonk, NY, USA).

#### Ethics

The GynOp register was approved by the Ethics Committee of the University of Umeå, Umeå, Sweden (Dnr 04–107). This study and the use of data from the register were approved by the Ethics Committee of the University of Umeå (Dnr 08–076 M).

## Results

**Fig. 1:**
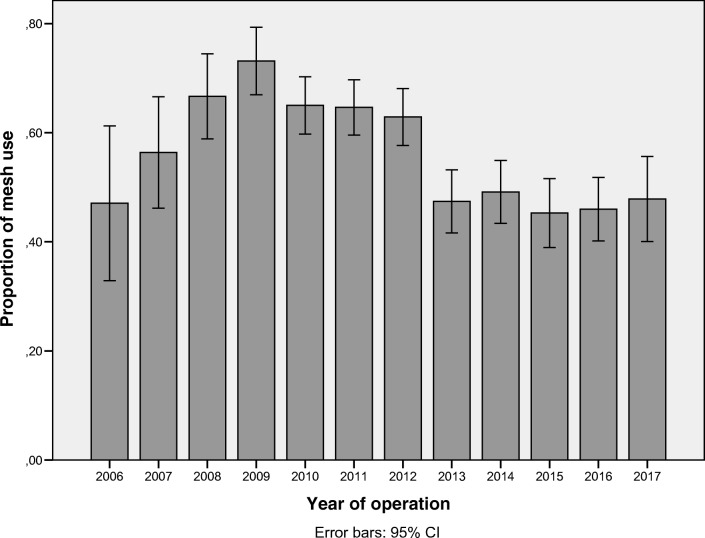
Proportions of mesh use in Sweden in operations for recurrent pelvic organ prolapse (POP) in the anterior compartment, 2006–2017; 95% CI = 95% confidence interval

The use of mesh in Sweden in operations for recurrent POP in the anterior compartment from 2006 to 2017 is shown in Fig. 1. At the national level, there was an increase in mesh use from 2006 to 2009 followed by a stable period, 2010–2012, at around 66% use. From 2013 (2 years after the FDA warning), there was a significant decrease (*p* = 0.000) to a new stable level of around 47%.

**Fig. 2:**
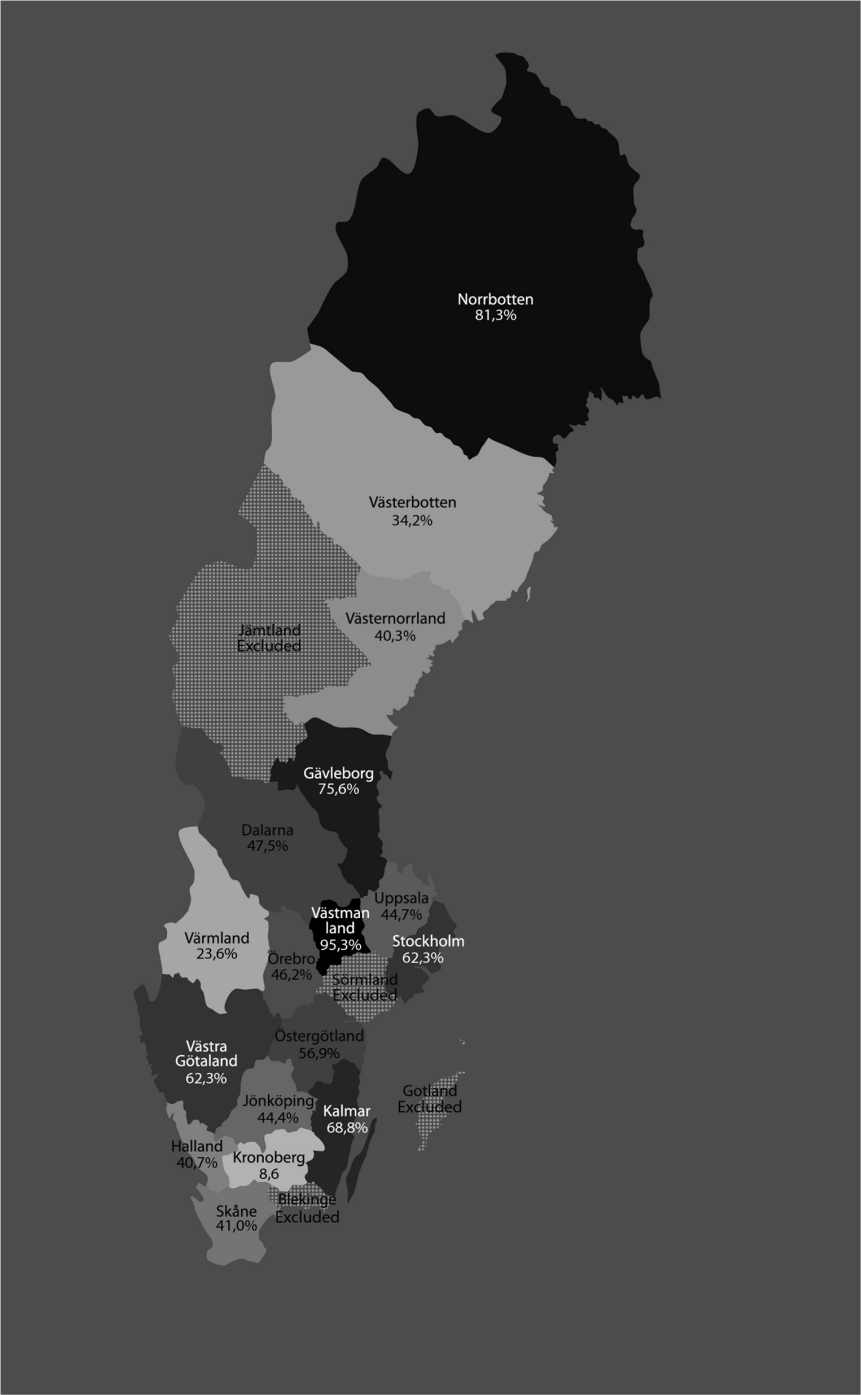
Aggregated use of mesh in Swedish counties in operations for recurrent pelvic organ prolapse (POP) in the anterior compartment, 2006–2017. Usage depicted in different shades of gray/black (light = low use; dark = high use)

**Fig. 3:**
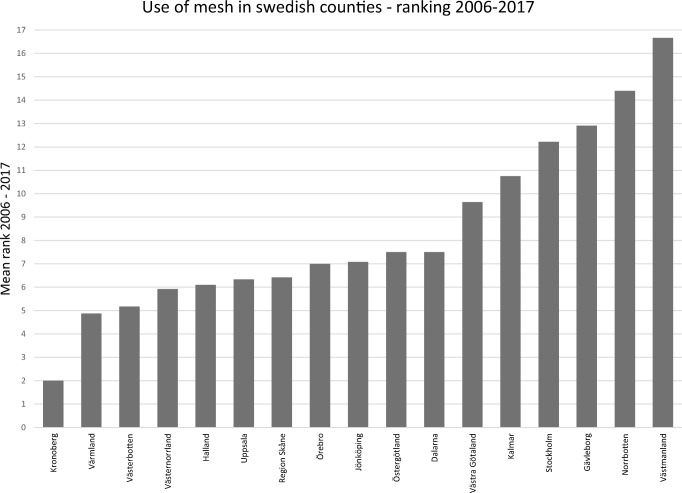
Mean rank of 17 Swedish counties regarding mesh use in operations for recurrent pelvic organ prolapse (POP) in the anterior compartment, 2010–2017. (The figure gives the annual ranking of the counties, where 1 = the lowest yearly rank in mesh use and 17 = the highest rank)

At a county level, however, the use of mesh varied significantly, with a range of 8.6–95.3%. Figure 2 shows aggregated proportions of mesh use of the 17 counties over the entire period studied. To examine possible concealed changes over time, we performed a yearly ranking of counties’ mesh use where 1 = the lowest yearly county rank in mesh use and 17 = the highest yearly county rank. Figure 3 shows the mean rank of the 17 Swedish counties from 2010 to 2017; it indicates that the ranking in mesh use has been fairly stable over time. We performed a logistic regression of decision to use mesh, with the year of procedure, county, and interaction between year and county as explanatory variables. This analysis showed that the interaction effect was insignificant (*p* = 0.732), but the main effects year (*p* = 0.000) and county (p = 0.000) were both significant, with a Nagelkerke R-square of 0.207. This substantiates that the use of mesh in the individual Swedish counties was independent of the year of surgery.

The patient pool was practically identical across the Swedish counties concerning age, body mass index (BMI), and number of births. The size of the prolapse, however, varied substantially among the counties (Table 1). To facilitate the evaluation, we here express the size of the prolapse as the distance from the lowest point of the prolapse to the hymen. Mean prolapse in the different counties ranged from 0.03 cm inside to 2.52 cm outside the hymen. A test of the relationship between prolapse size and mesh use was insignificant (*p* = 0.236). The different counties clearly had different strategies for deciding to perform the procedure with respect to the size of the prolapse, but this had no bearing on their propensity to use mesh. Differences in mesh use were not attributable to patient characteristics.

**Table 1:** Patient characteristics by county

	Age, years (mean)	95% CI, age, years	BMI (mean)	95% CI, BMI	Parity (mean)	95% CI, parity	Size of prolapse (cm)^a^	95% CI, size of prolapse
Dalarna	67.2	65.5–68.9	26.6	25.9–27.3	2.5	2.3–2.6	1.12	0.80–1.44
Gävleborg	67.3	66.1–68.4	26.7	26.3–27.2	2.5	2.4–2.7	−0.03	0.27–0.22
Halland	66.43	64.1–68.6	27.2	26.3–28.2	2.7	2.4–3.0	0.20	0.19–0.60
Jönköping	68.9	67.1–70.7	26.7	25.0–27.4	2.6	2.4–2.8	0.98	0.72–1.52
Kalmar	67.1	65.1–69.0	26.5	25.9–27.2	2.4	2.2–2.6	0.88	0.57–1.19
Kronoberg	67.3	64.6–70.6	26.1	25.2–27.2	2.6	2.3–2.9	0.39	0.12–0.79
Norrbotten	66.7	65.1–68.4	26.4	25.8–27.0	2.5	2.4–2.7	0.78	0.64–0.92
Region Skåne	67.1	65.8–68.4	26.4	25.9–26.9	2.5	2.3–2.6	1.16	−0.93–1.39
Stockholm	67.2	65.8–68.5	25.7	25.3–26.1	2.3	2.2–2.4	0.90	0.73–1.06
Uppsala	69.9	67.5–72.3	25.6	24.7–26.6	2.6	2.2–3.0	1.38	1.03–1.73
Värmland	67.3	64.4–70.2	26.5	25.9–27.1	2.4	2.2–2.5	0.44	0.23–0.66
Västerbotten	67.6	65.7–69.5	27.1	26.2–27.9	2.5	2.2–2.7	2.13	1.75–2.50
Västernorrl.	66.3	64.6–68.7	27.4	26.6–28.2	2.4	2.2–2.7	0.56	0.14–0.97
Västmanland	67.3	64.4–70.2	26.5	25.5–27.5	2.6	2.3–2.8	2.52	2.09–2.95
V. Götaland	66.9	65.9–67.9	26.2	25.9–26.6	2.5	2.4–2.7	0.96	0.82–1.10
Örebro	66.8	3.9–69.6	26.6	25.6–27.5	2.4	2.2–2.7	0.46	0.17–1.75
Östergötland	67.4	65.8–69.9	26.3	25.7–27.0	2.5	2.3–2.8	1.54	1.25–1.84

Characteristics of patients operated on for recurrent cystocele in Sweden in 2006–2017, stratified by county

*BMI* body mass index, *95% CI* 95% confidence interval

^a^Size of prolapse: distance from the lowest point of the prolapse to the hymen. Negative numbers indicate prolapse inside the introitus and positive numbers refer to prolapse outside the hymen

## Discussion

In this study, we analyzed 2758 consecutive POP operations over 12 years in comparable patients undergoing their first recurrent POP surgery in the anterior compartment. The strength of this material is its size and completeness. A weakness of non-randomized studies like ours is the risk of confounding because prolapse operations have many levels of difficulty, ranging from simple procedures in day surgery to very advanced operations with unsolved reconstructive problems. We tried to compensate for this by strict selection, resulting in a group of highly comparable patients, thus avoiding confounding by special anatomical or technical/operative necessities and enabling us to evaluate surgeons’ decision making.

At the national level, the use of mesh for recurrent cystocele has been fairly stable, giving an illusion of a certain consensus: in 2006–2009 (which can be interpreted as the learning period), there was a stepwise increase in mesh use followed by two stable rates of around 66% from 2009 to 2012 and around 47% from 2013 onwards.

Among the Swedish counties, however, the use of mesh differed by a factor of 11 (range 8.6–95.3%) in our observation period. The decision-making patterns in the individual counties remained the same from 2006 to 2017: Counties with low use of mesh kept having low use and counties with high use continued high use through all 12 years. The FDA warning led to a general decrease in mesh application, but the divergent pattern of mesh use prevailed.

Evidence-based decision making is one of the core values of any health care organization, and the choice between different treatment options is assumed to be a rational process. Based on this principle, the greater the amount of valid scientific information physicians receive, the more structured their beliefs should become and the more convergence it is reasonable to expect in their decision-making patterns when treating comparable patients [15–17].

A decade ago, when decisions regarding mesh use were hampered by limited evidence, different surgeons drew different conclusions from the available information. This has led to clear “communities of practice” at the county level regarding interpretation of existing scientific information about the effectiveness of mesh.

In the last decade, the amount of scientific information on the use of mesh in POP has increased enormously. A PubMed search for “(Pelvic organ prolapse AND (mesh OR implant))” in July 2018 yielded more than 2200 articles on the subject. A Cochrane review on transvaginal mesh compared with native tissue repair analyzed 37 randomized controlled trials of the intervention [18].

Since 2006, GynOp has distributed annual quality reports to all Swedish gynecological surgeons. The results are stratified according to the regional, county, and hospital level; consequently, the differences among counties are well known to the surgeons. Still, unaltered through 12 years, these groups have made mesh decisions in a clearly biased fashion, highly influenced by geographical factors, with unchanged disparity and with no measurable change toward consensus in the treatment of recurrent cystocele. It is not within the scope of this article to argue whether the use of mesh should be low or high. However, when the application of mesh ranges from 8.6 to > 95% in treatment of the same condition in comparable patients, the greater part of the underlying decisions must be suboptimal; the surgeons just cannot agree on which part.

The fact that Swedish surgeons’ decision-making patterns have remained unchanged, despite mounting information on the conditions under which mesh is useful or not, suggests that Swedish surgeons’ decisions may be attributable to two factors: (1) The available scientific information may not qualify, or be interpreted, as evidence and/or (2) surgeons may read scientific information selectively. In the case of POP surgery, where surgeons have worked on patients and drawn their own conclusions regarding the conditions under which mesh is useful or not, this may make them susceptible to favoring information that supports their own prior hypotheses. Whether one or a combination of both of the above factors is the underlying reason, the result is disturbing and unsettling.

A large disparity in surgeons’ decisions can be stimulating. It is an indication that there is potential for improvement and can be seen as a challenge to communicate and learn from each other. However, Swedish surgeons have maintained their contradictory positions for more than a decade with unchanged disparity. This indicates that the necessary scientific communication and learning process has stopped—despite the abundance of publications and the steady supply of new types of mesh to replace withdrawn ones [19].

For surgeons, this shows an astonishing mismatch between learning needs and learning readiness.

For patients, this represents 12 years of a geographical lottery concerning whether mesh is used or not.

The extraordinary disparity in mesh use between 15 OECD countries, shown in a 2012 survey, indicates that this is by no means a Swedish problem alone, but an international challenge [8].

To invigorate the surgical learning process, it seems prudent to question the apparently biased ways we glean evidence from the available information.

A sensible way forward would be to focus on increased communication across established consensus groups to enhance awareness of and curiosity about different solutions, increase willingness to learn from each other, and view differences as a possibility to learn and not a chance to dominate. In Sweden, this communication would need to take place between counties—in the OECD, between member countries.
